# Certification of a Family‐Friendly Intensive Care Unit—Families Are Always Welcome!

**DOI:** 10.1111/nicc.70074

**Published:** 2025-06-03

**Authors:** Antje Scheer, Michaela Denhof, Arnold Kaltwasser, Maria Kortgen, Sabina Mason, Sabrina Pelz, Peter Nydahl

**Affiliations:** ^1^ Deutsche Gesellschaft für Fachkrankenpflege Und Funktionsdienste eV Berlin Germany; ^2^ Department of Education for Skills Development in Healthcare Professions University Hospital Düsseldorf Düsseldorf Germany; ^3^ Representative North Rhine‐Westphalia and Spokesperson for the Ethics Working Group DGF eV Duesseldorf Germany; ^4^ Academy of the District Hospitals Reutlingen Reutlingen Germany; ^5^ Department of Educational Strategies for Healthcare Professions University Hospital Jena Jena Germany; ^6^ Board Member DGF eV Jena Germany; ^7^ Intensive Care Unit Tallaght University Hospital Dublin Ireland; ^8^ All Ireland Critical Care Research Nurses and Research Group Representative; Irish Research Nurses and Midwives Committee Member Dublin Ireland; ^9^ Intensive Care Unit University Hospital Tuebingen Tuebingen Germany; ^10^ EfCCNa Representative Germany of DGF Berlin Germany; ^11^ Nursing Research and Development University Hospital Schleswig‐Holstein Kiel Germany; ^12^ Institute of Nursing Science and Development Paracelsus Medical University Salzburg Austria

**Keywords:** critical care, family, humanizing, intensive care unit, patient‐centredness, patients

## Abstract

Involving family members in the daily care of intensive care unit (ICU) patients can increase family satisfaction and improve ICU patient outcomes. The presence of families in the ICU is a quality indicator and key factor for the recovery of patients in the ICU. The German Society for Specialised Critical Care Nursing's project “Family‐Friendly Intensive Care Unit” offers certifications to ICUs that implement flexible visiting hours and family‐centred care practices. A customizable points‐based system evaluates criteria such as educational offerings, architectural design and family involvement, thereby stimulating continuous quality improvement. This project might serve as a model for ICUs in other countries to implement holistic, evidence‐based care that supports patients, their families and healthcare staff.

Certification as a Family‐Friendly ICU includes the following: (1) Patient‐centred care: In the model “family‐friendly ICU” families and significant others are acknowledged and respected as an important part of a patient's life. (2) Enhanced family support: Policies that prioritize the emotional and psychological needs of families foster a supportive environment during critical care. (3) Improved communication: Clear and compassionate communication protocols are established between healthcare providers and families and ensure involvement in decision‐making. (4) Holistic care approach: integration of family‐centred practices leads to better patient outcomes and satisfaction for both patients and families. (5) Staff training and well‐being: training for staff on family engagement enhances their skills while supporting their emotional well‐being in high‐stress environments. (6) End‐of‐life care: family‐centred care approach can facilitate care planning and decision‐making during end‐of‐life situations.

Summary

- What is known about the topic
  ○ Family‐centred care (FCC) in the ICU improves patient recovery, reduces psychological burden for families, and enhances communication between healthcare teams and relatives.
  ○ Despite evidence and guidelines supporting open visiting hours and family integration, many ICUs still have restrictive policies, particularly in European countries.
  ○ Certification programs for family‐friendly ICUs can serve as a quality benchmark, encouraging structured implementation of evidence‐based, family‐centred practices.
- What this paper adds
  ○ This paper describes a structured, point‐based certification system developed by the German Society for Specialised Critical Care Nursing (DGF) to formally recognize family‐friendly ICUs.
  ○ It presents updated certification criteria co‐developed with ICU nursing experts, ensuring feasibility, adaptability, and alignment with current evidence and guidelines.
  ○ The paper offers a model for international adoption, highlighting how certification can stimulate cultural change, improve staff satisfaction, and promote sustained family‐centred care.

## 1 Background

The inclusion of family members in intensive care units (ICUs) has positive effects on patients, their families and the therapeutic team [1]. Families of ICU patients (including relatives, friends and significant others) find themselves in exceptional circumstances, are highly vulnerable, and require care and support [2]. During the COVID‐19 pandemic, it became evident that isolated patients and families without access to their loved ones faced increased psychosocial and psychiatric risks, underscoring the need for further research and compelling arguments to prepare for future pandemics [3, 4].

Family‐friendly care contributes to the humanization of intensive care medicine and is recommended by the updated Family‐centred care (FCC) guideline of the Society of Critical Care Medicine [5]. Involving families in the care of ICU patients can change challenging situations to more comprehensible and bearable caring for everyone involved [1]. Integrating family members can minimize the risks of post‐traumatic stress conditions, such as post‐intensive‐care syndrome (PICS), for both patients and their relatives [6, 7, 8]. Furthermore, family integration has been associated with reduced risks of anxiety, agitation, delirium and increased satisfaction and shorter hospital stays [9, 10, 11].

Over the past two decades, the concept of FCC in ICUs has evolved from restrictive visiting policies and a largely theoretical ideal to a recognized component of quality care [12]. Early discussions in the mid‐2000s questioned the practical challenges of implementing FCC and highlighted the need to better acknowledge the experiences and needs of families [13]. Yet, even a decade later, these restrictive visiting policies and limited family involvement remained widespread in many European ICUs, despite growing evidence of the positive impact of family presence on patient outcomes and satisfaction [14]. In response to this persistent gap between evidence and practice, a structured initiative was launched in 2024 by the German Society for Specialised Critical Care Nursing (Deutsche Gesellschaft für Fachkrankenpflege und Funktionsdienste eV, DGF). This initiative offers formal certification for ICUs that fulfil a set of clearly defined criteria, recognizing them as family‐friendly units and promoting sustainable integration of FCC principles into everyday care.

### 1.1 FCC as a Benchmark of Quality

The integration of family members as part of the interprofessional team is increasingly recognized as a quality indicator in modern intensive care medicine and nursing [15, 16]. Family‐centred approaches and support from familiar caregivers enhance the emotional stability of patients. In addition to the practical assistance provided by family members, such as facilitating communication processes, their presence fosters a sense of care and security which is a key factor in the recovery process. To achieve this, family members must have the opportunity to be present in the ICU, which necessitates flexible and open visiting hours. The updated SCCM guideline strongly recommends implementing liberalized ICU family presence policies as the standard practice in ICUs [5]. A certification of family‐friendly ICU might increase awareness and implementation.

### 1.2 Aim

The aim of the certification of a Family‐Friendly ICU is to promote the integration and recognition of family members as an essential component of patient‐ and FCC and high‐quality intensive care nursing. The aim of the DGF's project is to increase awareness, knowledge and improvement of FCC in practice.

In an international context, this description of the certification process can serve as a template for intensive care staff to initiate and support similar certification processes in their countries.

## 2 Methods

The German Nursing Foundation “Stiftung Pflege” launched the Family‐Friendly ICU project in 2008 and has since certified 250 ICUs in Germany. In the last 18 months, the DGF added 70 more certified ICUs. The certification grants family members the right to be with patients around the clock (24/7).

In 2024, the DGF continued and updated the project to support ICU staff in implementing these principles and to recognize their efforts through certification. Current literature was reviewed and aligned with available guidelines. As part of this process, existing certification criteria were revised to current conditions and new criteria developed. The main update was to establish a point‐based scoring system for achieving the approval. The criteria were finalized in collaboration with a team of nursing experts (*n* = 17) during eight virtual meetings. All nursing experts evaluated the feasibility of the criteria within their institutions.

Additionally, a website was created, providing a comprehensive description of the certification process, including costs, along with forms for applications (https://www.dgf‐online.de/angehoerigenfreundliche‐intensivstation).

### 2.1 Certification Process for Family‐Friendly ICU

The certificate “Family‐Friendly Intensive Care Unit – Families Are Always Welcome!”, initiated by the DGF, encourages hospitals to translate scientific evidence into practice. The process includes the following steps:

1. Application Submission: Submit the application via email or post to the DGF.
2. Content Review: Evaluation of the submitted documents by a person appointed by the DGF.
3. Audit and Goal Setting: If necessary, conduct an on‐site visit or online audit and define goals to be achieved within the following certification period of two, resp. 3 years.
4. Certificate Issuance: Validity: 2 years for initial certifications, 3 years for re‐certifications. The certificate is provided physically or digitally, optionally accompanied by personalized services.

### 2.2 Certification Criteria

Generally, all responsible personnel of the ICU must sign the certification application, including the leadership up to the hospital's board (directorate of nursing, medical directorate, executive management, patient representative). This facilitates the process and helps to prevent interprofessional conflicts.

A points‐based system has been developed for certification as a “Family‐Friendly Intensive Care Unit” to provide ICUs with flexible conditions and opportunities for tailored development. To achieve initial certification, a minimum of 30 points must be achieved. For re‐certification after 2, or 3 years, additional five points are required to support an ongoing developmental process. Two mandatory criteria, each worth 10 points, must be fulfilled: (a) flexible visiting hours within at least a 12‐h timeframe and (b) an appropriate waiting area for family members.

Additional criteria can be selected from a catalogue of 35 options, totalling up to 70 points, and are categorized as follows:

- Processes (e.g., proactive family calls, documentation of visits): 6 criteria, 12 points.
- Children's visits (e.g., encouraging visits from children of all ages): 4 criteria, 6 points.
- Written policies (e.g., informational brochures, protocols, SOPs on equality and inclusion): 4 criteria, 10 points.
- Support and integration opportunities for families (e.g., involving family members in decision‐making, ICU diaries, participation in ward rounds): 9 criteria, 25 points.
- Educational interventions for the team (e.g., at least two training sessions on the topic in the past year): 5 criteria, 10 points.
- Architecture (e.g., meeting rooms for discussions with family members): 3 criteria, 3 points.
- Support for families (e.g., meal options, restroom facilities for families on the ICU): 3 criteria, 3 points.
- Other (e.g., peer review): 1 point.

Criteria with guideline recommendations or strong evidence/relevance were assigned three points, while all other criteria received one point, to encourage the implementation of evidence‐based family care practices (Table 1). The complete criteria catalogue can be found in the appendix (Data S1).

**TABLE 1:** Example of criteria and points required for certification.

Criteria	Proof	Pt.^a^
Mandatory: Variable visiting hours	Flyer, Website, PDF	10
Mandatory: Waiting area, available exclusively for families^b^ on or in front of the intensive care unit	Flyer, Website, PDF	5–10
Processes		
24‐h possibility to call with telephone number	Website, Calling card or similar.	3
Proactive family phone call	Flyer, Website, PDF	1
Children's visits		
Visits from children are welcome	Information on website, flyer, door	3
Waiting area child‐friendly	Furniture for children, photo	1
Written concepts		
Information for families	Flyer, Website, PDF	3
Written concept of coordinated information for relatives with literature references	PDF	3
Support and integration services for families		
Offers for integration into care	Information sheet on integration, e.g., combing hair, applying body lotion, etc.; Website, PDF	3
Volunteer work	Flyer, Website, PDF with contact options	1
Training activities of the team		
At least one training course in the last 2 years on communication training with relatives, e.g., VALUE	List of participants	3
At least two educational trainings about Family‐Centred Care in the last year	List of participants	3
The intensive care unit		
Accessibility for caregivers with special needs (sufficiently wide doors for wheelchairs, sufficiently large toilets etc.)	Photo	1
Care of caregivers		
Overnight stay in/at the hospital and/or in the room	Photo	1

^a^ A total of 30 points must be achieved. The first two criteria are mandatory and therefore receive 10 points, resp. depending on the location of the waiting area 5–10 points. Guideline or evidence‐based criteria are assessed with 3 points, all other criteria with 1 point.

^b^ “Families” includes relatives, friends and others.

### 2.3 Example

A surgical ICU with 16 beds applies for certification at the DGF. The ICU offers visiting hours from 8:00 AM to 8:00 PM, with additional visits possible by individual arrangement (=10 points). A waiting area is available for families in a specific room with closable doors in the ICU (=10 points). The unit has a frequent educational programme, including (a) at least two training sessions for all staff in the past year on aspects of FCC (=3 points in total), (b) one session on the VALUE concept (Value family statements, Acknowledge emotions, Listen, Understand the patient as a person, Elicit questions) (=3 points) and (c) a collection of 10 “One‐Minute Wonders” on the subject (=3 points). Additionally, they implement a 24‐h call option with the phone number listed on the unit's website and business cards (=3 points). This brings their total to 32 points, qualifying them for certification as a Family‐Friendly ICU. Thanks to their high level of education on family‐friendly intensive care practices, the team discusses barriers and conflicts, finds compromises and solutions. After certification, the ICU team is awarded as best‐practice ICU in the hospital and local newspapers by nursing and hospital managers via websites, social media, newsletters and others. The journey continues, and the leadership plans to achieve an additional five points by the re‐certification in 3 years by implementing ICU diaries (three points) and involving family members in decision‐making processes (three points).

## 3 Results

Based on the feedback of certified ICUs, the process of certification as a Family‐Friendly ICU serves not only as an incentive to continuously improve the quality of care for patients and their loved ones but also contributes to optimizing staff working conditions. It promotes positive changes within the team by facilitating interactions with family members, enhancing communication and fostering a supportive work environment. Ultimately, these improvements positively impact employee satisfaction and motivation. Nevertheless, the project comes not without conflicts, and some staff might be reluctant to welcome families all day long. These conflicts shall be discussed and reflected on by the leadership and feasible solutions sought, for example, by a stepwise implementation and evaluation, feedback by families and patients and frequent educational meetings (Table 2).

**TABLE 2:** Barriers and solutions to family‐centred care.

Barriers	Solutions
Assumption, open visiting times would impede staff from working	Inform about real visiting times 2–4.5 h of families in 24/7 open ICU. Exception for very few patients under special conditions (dying, delirium, dementia), but here families^a^ help staff!
Families disturb working by asking so many questions and having needs	Use a proactive approach and ask families for their questions and needs when you want. Otherwise recognize their questions and needs and refer to later timepoint.
Cultural and organizational Barriers	Conduct training sessions to raise awareness of family‐centred care. Hold regular interdisciplinary meetings to foster cultural change.
Time and resource Constraints	Develop policies for interactions with relatives. Hire additional staff, such as social workers. Implement efficient shift scheduling to optimize time resources.
Inadequate spatial arrangements	Set up family rooms or retreat areas. Integrate virtual meeting tools for digital communication with families. Invest in the spatial redesign of intensive care units.
Emotional strain on staff	Organize workshops on emotional resilience. Implement a supervision system for healthcare workers. Promote team discussions to share emotional burdens.
Communication problems	Develop standardized conversation guides for communication with families. Provide regular updates via digital platforms. Conduct communication training for the team.
Data protection and legal barriers	Address data protection issues through expert lectures. Introduce consent forms for discussions with families. Regularly review data protection policies through an internal committee.
Divergent expectations of families	Hold informative sessions to manage expectations. Involve relatives in multidisciplinary case discussions. Provide informational materials about caregiving.
Nurses have limited time resources to support primary caregivers.	Discussion within the team to develop solutions that enable nurses to allocate time resources for advising and supporting families when needed.
Nurses show little understanding for open visiting hours	Team communication and training opportunities on the significance of open visiting hours in the intensive care unit for those affected.

^a^ “Families” includes relatives, friends and others.

Since the start of the project, more than 300 ICUs in Germany have been certified as Family‐Friendly ICU, representing one fifth of approximately 1500 ICUs in Germany. A survey on family‐friendly care in German‐speaking countries, including Germany, Austria and Switzerland, revealed that significantly more ICUs in Germany had open visiting hours. This outcome might be attributed, to some extent, to the Family‐Friendly ICU project [17]. The survey will be repeated this year to evaluate the cultural development.

## 4 Conclusions

The “Family‐Friendly Intensive Care Unit” project promotes patient‐ and family‐centred intensive care nursing, respecting and integrating the needs and wishes of everyone involved in the care process. The certification emphasizes the importance of holistic care and establishes evidence‐based standards. Hospitals are encouraged to adopt this approach actively and position themselves as pioneers in modern intensive nursing care.

## Ethics Statement

Due to the character of this publication, this work did not require an ethics approval.

## Consent

The authors have nothing to report.

## Conflicts of Interest

The authors declare no conflicts of interest.

## Supporting information


**Data S1.** Supporting Information.

## Data Availability

Data sharing is not applicable to this article as no new data were created or analyzed in this study.
