# Supplementary material for: Certification of a Family‐Friendly Intensive Care Unit—Families Are Always Welcome!
Source: Nurs Crit Care. 2025 Jun 3;30(4):e70074. doi: 10.1111/nicc.70074 (PMC12131286; doi:10.1111/nicc.70074)
Supplement: Supplementary file 1 — Data S1. Supporting Information. [file NICC-30-0-s001.docx]

**SUPPLEMENT**


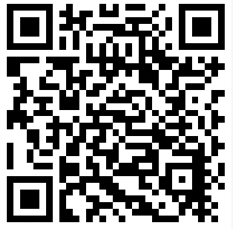
**Certification as a Family-Friendly Intensive Care Unit**

This supplement serves as a template for developing certification projects. The certification forms described here cannot be used to apply for certification abroad through the DGF.

Applications in German are available at: https://www.dgf-online.de/angehoerigenfreundliche-intensivstation/

### Implementation of the Criteria

The implementation of the specified measures is supported by structured processes and clear documentation requirements. These include regular feedback forms, audits, and adherence to the goals set within the certification period.

### Costs and Services

The costs for certification are €250 for initial certification and €200 for certificate renewal (re-certification) in 2024. Additional services, such as an acrylic sign or the personal delivery of the certificate, can be added at extra cost.

### Criteria Catalogue for Family-Friendly Intensive Care Units

#### Criteria

| **Criteria** | **Evidence** | **Points** |
| --- | --- | --- |
| **Mandatory Field: Flexible Visiting Hours** | Flyer, Website, PDF | 10 |
| **Mandatory Field: Dedicated Waiting Area for Family Members on or near the ICU** | Flyer, Website, PDF | 5-10* |

#### *depending on location (in/near ICU), presence of doors (y/n), and number of (artificial) walls (at least 3 walls; one wall would be waiting area in the corridor and this is not accepted)

#### Processes

| **Criteria** | **Evidence** | **Points** |
| --- | --- | --- |
| 24-hour Call Option with Phone Number | Website, Business Card, etc. | 3 |
| Proactive Family Calls | Flyer, Website, PDF | 1 |
| Interactive Discussions Guided by Protocols | PDF | 3 |
| Scheduled Appointments for Family Members | Flyer, Website, PDF | 1 |
| Documentation of Family Presence | Documentation | 1 |
| Documentation of Discussion Contents and Agreements with Family Members | Documentation | 3 |

#### Children’s Visits

| **Criteria** | **Evidence** | **Points** |
| --- | --- | --- |
| Children’s Visits Encouraged | Information on Website, Flyer, Door | 3 |
| Concept for Welcoming Child Visitors in Place | PDF | 1 |
| Information Available for Children | Flyer, Colouring Books | 1 |
| Child-Friendly Waiting Area | Child-Sized Furniture, Photo | 1 |

#### Written Policies

| **Criteria** | **Evidence** | **Points** |
| --- | --- | --- |
| Information for Family Members | Flyer, Website, PDF | 3 |
| Written Concept for Coordinated Family Information, Including Literature References | PDF | 3 |
| Policies for Supporting Non-German-Speaking Patients and Families (e.g., Interpreter Availability, Translator Apps) | PDF | 1 |
| Equality and Inclusion Policy Document Addressing Equal Treatment Regardless of Social, Professional, Educational Backgrounds, Nationalities, Language Abilities, and Other Factors | PDF | 3 |

#### Support and Integration Offers for Family Members

| **Criteria** | **Evidence** | **Points** |
| --- | --- | --- |
| Involvement of Family Members in Decision-Making Processes | Documentation | 3 |
| ICU Diaries Offered | Diary, Website, PDF | 3 |
| Opportunities for Family Members to Participate in Care Activities (e.g., Combing Hair, Applying Cream) | Information Sheet, Website, PDF | 3 |
| Grief Counselling Services Offered | Flyer, Website, PDF | 3 |
| Psychosocial Support, Including Mental Health Assessments and Training in Coping Strategies | Flyer, Website, Contact Details, PDF | 3 |
| Spiritual Support Services Considering Cultural and Religious Diversity | Flyer, Website, PDF | 3 |
| Family Presence Permitted During Procedures (e.g., Central Line Placement, CPR) Upon Agreement | Documentation, Flyer, Website, PDF | 3 |
| Family Attendance During Ward Rounds | Flyer, Website, PDF | 3 |
| Use of Volunteer Staff | Flyer, Website, PDF with Contact Information | 1 |

#### Team Training Measures

| **Criteria** | **Evidence** | **Points** |
| --- | --- | --- |
| At Least Two Staff Training Sessions on Family-Friendly ICU Practices in the Past Year | Attendance List | 3 |
| At Least One Communication Training Session (e.g., VALUE) in the Past Two Years | Attendance List | 3 |
| At Least One Training Session in the Past Two Years on Guiding/Training Family Members for Patient Care | Attendance List | 1 |
| At Least One Training Session in the Past Two Years on Digital Communication Tools | Attendance List | 1 |
| Use of "One-Minute Wonders" on Family-Friendly ICU Practices | One-Minute Wonders Documentation | 1 |

#### The Intensive Care Unit

| **Criteria** | **Evidence** | **Points** |
| --- | --- | --- |
| Meeting Room for Discussions with Family Members | Photo | 1 |
| Accessibility for Family Members Requiring Mobility Aids (e.g., Wheelchair-Accessible Doors, Adequate WC Facilities) | Photo | 1 |
| Family-Oriented Interior Design (e.g., Clear Signage, Easily Understandable Room Numbers, Accessible WC Facilities) | Photo | 1 |

#### Services for Family Members

| **Criteria** | **Evidence** | **Points** |
| --- | --- | --- |
| Self-Service Meal Options on the Ward/In the Waiting Area or Provided by Staff Upon Request, or in the Cafeteria | Photo | 1 |
| Overnight Accommodation Within or Near the Hospital or in Patient Rooms | Photo | 1 |
| WC Facilities for Family Members on the Ward | Photo | 1 |

#### Miscellaneous

| **Criteria** | **Evidence** | **Points** |
| --- | --- | --- |
| DIVI Peer Review Conducted in the Past Two Years | Report | 1 |

A total of 30 points must be achieved. The first two criteria are mandatory and each carry 10, resp. 5-10 points. Criteria based on guidelines or evidence are awarded 3 points, while all other criteria receive 1 point.
